# Pragmatic language dysfunction in systemic lupus erythematosus patients: Results from a single center Italian study

**DOI:** 10.1371/journal.pone.0224437

**Published:** 2019-11-04

**Authors:** Fulvia Ceccarelli, Carmelo Pirone, Concetta Mina, Alfredo Mascolo, Carlo Perricone, Laura Massaro, Francesca Romana Spinelli, Cristiano Alessandri, Guido Valesini, Fabrizio Conti

**Affiliations:** 1 Lupus Clinic, Dipartimento di Medicina Interna e Specialità Mediche, Sapienza University of Rome, Rome, Italy; 2 Dipartimento di Neurologia e Psichiatria, Sapienza Università di Roma, Rome, Italy; 3 Clinica Neurologica, Dipartimento di Neuroscienze, Università degli studi di Roma Tor Vergata, Rome, Italy; Keio University, JAPAN

## Abstract

**Background:**

Cognitive impairment (CI) in systemic lupus erythematosus (SLE) is a frequent neuropsychiatric manifestation affecting several domains, even in apparently asymptomatic patients. Current research revealed that the typical CI pattern affects frontal-subcortical circuit and thus executive functions. The impairment of non-literal language or pragmatic language (PL), including metaphors, idioms, inferences or irony has been well described in several conditions such as autism disorders, Parkinson’s disease, brain injury and even in earlier phases of neurodegenerative processes. Even if PL neuro-anatomy remains controversial, correlation between executive dysfunctions and non-literal language involvement has been reported both in traumatic injury and mild cognitive impairment patients. Nonetheless, no specific study has been performed to evaluate PL impairment in SLE patients so far.

**Objectives:**

We aimed at assessing the PL domain in a Italian monocentric SLE cohort in comparison to healthy controls, matched to age and education, through a specific battery, the *batteria sul linguaggio dell'emisfero destro* (BLED). Secondly, we focused attention on possible correlations between CI and clinical and laboratory SLE-related features.

**Methods:**

Forty adult patients affected by SLE, according to the American College of Rheumatology (ACR) criteria, and thirty healthy subjects were enrolled consecutively in this cross-sectional study. The protocol included complete physical examination, extensive clinical and laboratory data collection (comprehensive of demographics, past medical history, co-morbidities, disease activity, chronic damage evaluation, previous and concomitant treatments) and cognitive assessment for five different domains: memory, attention, pragmatic language, executive and visuospatial functions. Self-reported scale for anxiety and depression were performed to exclude the influence of mood disorders on cognitive dysfunction.

**Results:**

We studied 40 Caucasian SLE patients [male (M)/ female (F) 3/37; mean±standard deviation (SD) age 45.9±10.1 years, mean±SD disease duration 120.8±81.2 months] and 30 healthy subjects (M/F 9/21; mean±SD age 41.3±13 years). According to the low level of disease activity and damage (mean±SD Systemic Lupus Erythematosus Disease Activity Index 2000 (SLEDAI-2K) of 1.3±2.3, mean±SD Systemic Lupus International Collaborative Clinics/American College of Rheumatology (SLICC/ACR) Damage Index (SDI) of 0.2±0.5), only 30% of patients was on glucocorticoid treatment at the study entry. PL was the most compromised domain in terms of Mean Domain Z scores. As for the Domain Cognitive Dysfunction score, a deficit of PL was observed in 45% of patients and was significantly more prevalent than memory, executive and visuospatial functions impairment (P = 0.0002, P = 0.0002 and P<0.000001, respectively). According to Global Cognitive Dysfunction score, 25% of patients experienced a mild impairment and 7.5% a moderate one. Anti-phospholipid antibodies positivity was significantly associated with memory impairment (P<0.0005), whereas the presence of other neuropsychiatric events was associated with executive dysfunctions (P<0.05); no further significant association nor correlation were identified.

**Conclusion:**

In this study we evaluated for the first time PL in SLE patients finding a dysfunction in almost half of patients. The dysfunction of PL was significantly more frequent than the other domains assessed.

## Introduction

Cognitive impairment (CI) in Systemic Lupus Erythematosus (SLE) is a frequent neuropsychiatric manifestation occurring in up to 90% of patients [1,2]. Neurocognitive test battery often highlights deficit of cognitive domains widely ranging from memory, language and motor dexterity to executive functions, attention, visuospatial skills, verbal and non-verbal fluency, even in patients without overt neuropsychiatric SLE (NPSLE) [3,4]. The extensive spectrum of CI has been likely ascribed to a broad variety of pathogenetic mechanisms affecting nervous system (e.g. vasculopathy, coagulopathy, autoantibodies and cytokine-mediated neuronal dysfunctions through blood-brain barrier damage) [5]. Nonetheless, recent research has revealed a most typical CI pattern in SLE patients involving fronto-subcortical region of brain suggested by the abnormal activation in the frontal cortex observed by functional Magnetic Resonance Imaging (MRI) and by the correlation between SLE-related CI and white matter hyperintensities [6,7].

To date, impairment of non-literal language, including metaphors, idioms, inferences, or irony has been well described in several conditions such as autism disorders, schizophrenia, Parkinson’s and Alzheimer’s diseases, right hemisphere traumatic lesions, and early phases of neurodegenerative processes [8].

Non-literal language—or so-called pragmatic language (PL)—is the ability of understand expression used in real-world situations beyond the strictly literal speech [9]. Even if PL neuro-anatomy remains controversial, a recent meta-analysis indicates that a predominantly left lateralized network, including frontal, temporal, para-hippocampal and prefrontal cortex, is pathogenetically relevant [10]. Several studies suggested the role of specific executive functions in the PL understanding [11, 12]. Moreover, a correlation between executive impairment and difficulties in pragmatic communication have been reported both in traumatic injury and mild cognitive impairment [13,14].

Despite the high frequency of executive functions impairment detectable in SLE patients, no specific studies evaluated PL impairment in these patients so far.

Thus, we performed a cross-sectional study to assess the PL domain in a monocentric cohort of SLE patients using a specific neurocognitive scale—*Batteria sul Linguaggio dell'Emisfero Destro* (BLED) -, to evaluate non-literal comprehension.

## Patients and methods

We enrolled consecutive Caucasian Italian adult SLE patients followed up at Lupus Clinic, Sapienza University of Rome, fulfilling the American College Rheumatology (ACR) revised classification criteria for SLE [15]. As control, we enrolled healthy subjects (HS), matched for age and education level. The local ethic committee of Policlinico Umberto I–Sapienza University of Rome approved this study, conducted according to the principles expressed in the Declaration of Helsinki. A written informed consent was obtained from patients and HS before the enrollment.

According with the study protocol, SLE patients underwent complete physical examination; clinical and laboratory data were collected in a standardized computerized electronically filled form including demographics, past medical history, co-morbidities, previous and concomitant treatments. Antinuclear antibodies (ANA) were determined by indirect immunofluorescence assay (IIFA) on HEp-2, anti-dsDNA by IIFA on Crithidia luciliae, ENA (anti-Ro/SSA, anti-La/SSB, anti-Sm, anti-RNP), anti-cardiolipin (anti-CL) of IgG or IgM isotype and anti-Beta2glicoprotein I (anti-Beta2GPI) of IgG or IgM isotype by ELISA. Lupus anticoagulant (LA) was assessed according to the guidelines of International Society on Thrombosis and Hemostasis. For all the subjects, complement C3 and C4 concentrations were determined by nephelometry.

Disease activity was assessed by using the SLE Disease Activity Index 2000 (SLEDAI-2K) and the chronic damage by the Systemic Lupus International Collaborative Clinics/American College of Rheumatology (SLICC/ACR) Damage Index (SDI) [16, 17].

Patients and controls underwent an extensive cognitive assessment during a 1-hour interview including standardized testing for five domains: memory, attention, pragmatic language, executive and visuospatial functions. This assessment included the tests from ACR and the Cognitive Symptom Inventory (CSI) standardized in an Italian population, specifically designed to detect the fronto-subcortical dysfunction typical of SLE and PL dysfunctions [1, 18, 19]. Beck Depression Inventory II and Zung self-assessment questionnaires for anxiety were administered to exclude the influence of mood disorders on cognitive dysfunctions [20,21]. The following tests were used: Mini Mental State Examination for general cognitive status (MMSE) [22]; Digit Span forward and backward, an efficient neuropsychological instruments for testing verbal memory; Attentive Matrices for both selective and sustained attention; the Rey–Osterrieth complex figure test to assess visuospatial functions; Phonological Verbal Fluency Test, Trail Making Test A, Trail Making Test B (to investigate deeply the presence of executive dysfunctions) [23,24].

Moreover, the BLED Santa Lucia battery was applied to assess non-literal language through four different tests: irony, metaphors, inferences and requests [25].

### Statistical analysis

Unadjusted analysis was performed as previously described [4]. Briefly, for each patient, the raw scores from each test were compared with published norms (age-, sex-, and education level-corrected, when necessary) and transformed into Z scores to express the deviation from the normal mean [Z = (raw data-test mean)/test standard deviation]. Mean domain Z scores (MDZs) were defined as the average of the Z scores from the tests comprising each domain. To indicate cognitive function as a composite score, the Z score for each domain was transformed into a Domain Cognitive Dysfunction score (DCDs), with higher values representing greater impairment in a given domain. The sum of all DCDs across the five domains resulted in the Global Cognitive Dysfunction score (GCDs), which was transformed into a Global Cognitive Dysfunction category (GCDc). The results obtained from healthy controls evaluation were applied to calculate Z scores for pragmatic language battery only, as mentioned above, because of the lack of reference ranges for this age group.

The statistical analysis was performed with GraphPad 5.0 (La Jolla, CA, USA). Normally distributed variables were summarized using the mean±standard deviation (SD), and non-normally distributed variables by the median and interquartile range. Wilcoxon’s matched pairs test and paired t-test were performed. Univariate comparisons between nominal variables were calculated using chi-square (x^2^) test or Fisher-test where appropriate. Two-tailed P values were reported, P values less than or equal to 0.05 were considered significant. GCDs were compared in patients grouped by antibody level. The binary outcomes variable for the antibody testing were serum autoantibody status, defined either as present versus absent or low/absent versus high. The results were verified through analysis of the domain Z scores and single-test Z scores. Descriptive statistics were computed for all study variables. Multivariable logistic regression analysis was performed including only variables that achieved P value < 0.100 in the univariate analysis were included for calculation.

## Results

We enrolled 40 Caucasian SLE patients [male(M)/female(F) 3/37; mean±SD age 45.9±10.1 years; mean±SD disease duration of 120.8±81.2 months] and 30 HS (M/F 9/21; mean±SD age 41.3±13 years) The general cognitive status was in the range of normality in the whole cohort, as shown by mean MMSE levels of 29.3±1.2 (mean±SD). Table 1 summarizes the main clinical and demographic features of the cohort, including ongoing therapy.

**Table 1:** Clinical and demographic features of SLE patients (N = 40) enrolled in the study.

**ACR criteria**	**N/%**
Malar rash	21/52.5
Discoid lupus	1/ 2.5
Photosensitivity	17/42.5
Mouth and nasal ulcers	29/72.5
Arthritis	29/72.5
Serositis	8/20
Renal involvement	8/20
Hematologic manifestations	20/50
Neuropsychiatric involvement	5/12.5
**Autoantibodies**	**N/%**
Anti-dsDNA	29/72.5
Anti-SSA	11/27.5
Anti-SSB	4/10
Anti-SM	6/15
Anti-RNP	5/12.5
Hypocomplementemia	19/47.5
Anti-cardiolipin	13/32.5
Anti B2GPI	11/27.5
Lupus Anticoagulant	8/20
**Therapy**	**N/%**
Glucocorticoids	12/30
Hydroxychloroquine	29/72.5
Methotrexate	6/15
Azathioprine	12/30
Cyclosporine	5/12.5
Mycophenolate mofetil	6/15
Cyclophoshamide	1/ 2.5
Belimumab	1/2.5
Rituximab	2/5
Antiplatelet therapy	12/30
Anticoagulant treatment	2/5

The cohort showed a mean±SD SLEDAI-2K of 1.3±2.3 and mean±SD SDI 0.2±0.5; according to the low disease activity and damage scores, only 30% of patients was on glucocorticoid treatment when evaluated.

Regarding concomitant autoimmune diseases, six patients (15%) were affected by anti-phospholipid syndrome (APS) and three (7.5%) by Sjögren’s syndrome. Furthermore, cardiovascular risk factors were reported as follows:: arterial hypertension in 10 patients (25%), diabetes in one (2.5%) and dyslipidemia in 4 (10%). Finally, thyroid disease was observed in 8 patients (20%).

**Fig 1:**
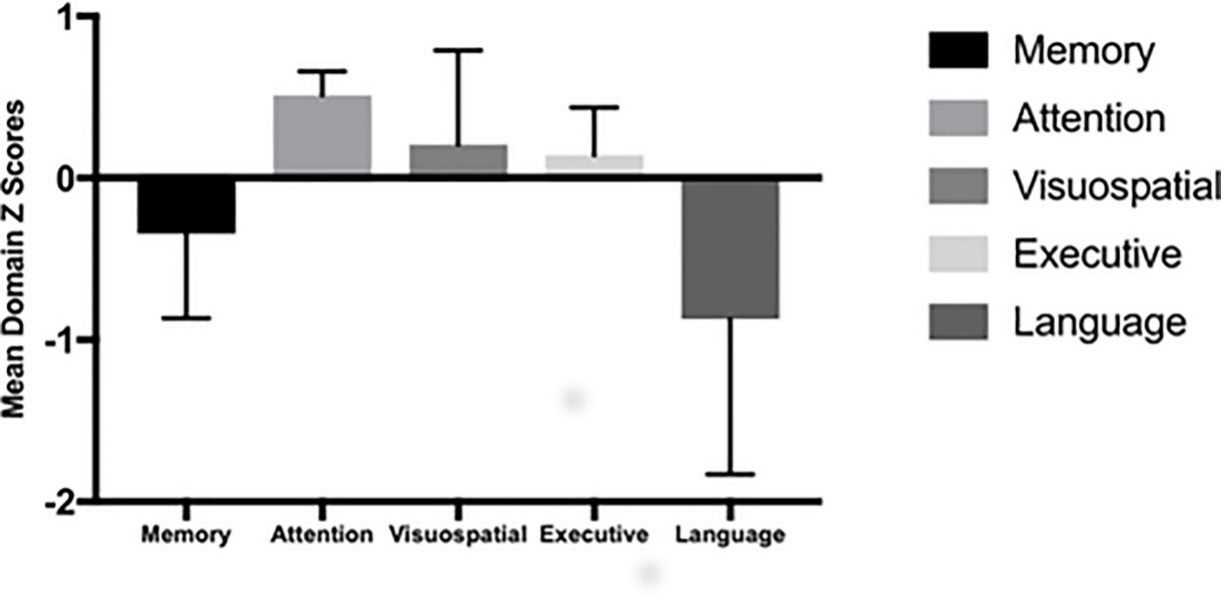
Distribution of neurocognitive impairment expressed in MDZs among the SLE patients enrolled (N = 40).

When considering the patients’ Z domain scores, PL was the most compromised [Memory: median -0.34 (IQR 1.11); Attention: median 0.51 (IQR 0.42); Visuospatial: median 0.21 (IQR 1.02); Executive: median 0.14 (IQR 0.89); Language: median -0.87 (IQR 1.71)] as shown in Fig 1.

Table 2 summarizes the percentage of patients with CI in the different domains after transforming the MDZs into DCDs.

**Table 2:** Percentage of patients with grade 0 to 2 CI, expressed as DCDs, in the different domains.

	Memory (N/%)	Executive (N/%)	Attention (N/%)	Visuospatial (N/%)	Language (N/%)
*DCDs =* ***0***	32/80	33/82.5	40/100	37/92.5	22/55
*DCDs =* ***1***	8/20	5/12.5	0	3/7.5	9/22.5
*DCDs =* ***2***	0	2/5	0	0	9/22.5

DCDs: Domain Cognitive Dysfunction score

Noticeably, a deficit of PL was observed in 45% of patients and was significantly more prevalent than memory, executive and visuospatial functions impairment (P = 0.0002, P = 0.0002 and P<0.000001, respectively).

**Fig 2:**
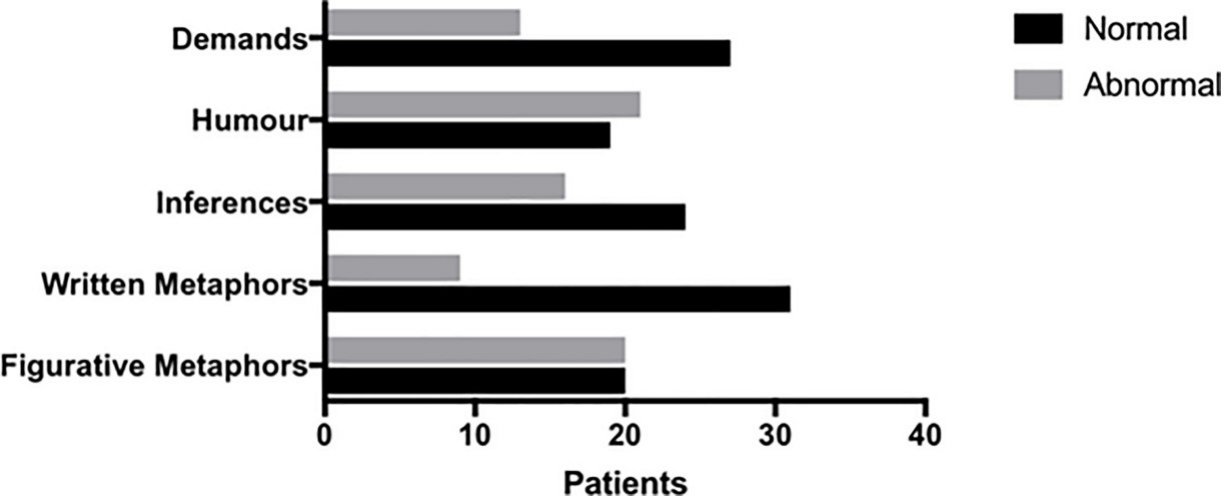
Prevalence of BLED Santa Lucia test dysfunctions among the SLE patients enrolled (N = 40) according to MDZs.

Considering the general cognitive impairment, 25% of patients experienced a mild impairment (GCDs 2–3), 7.5% moderate (GCDs 4–5). None of patients had a severe impairment. In detail, Fig 2 highlights dysfunctions of language specific tests.

Among the PL impairment assessed in our cohort, humor, figurative metaphors and inferences were the most frequently compromised skills (52.5%, 50% and 40%, respectively), as reported in Fig 2.

According to self-reported scales, a depressive status was identified in 22 patients (55%) and anxiety in about the totality of the cohort enrolled (92.5%). However, when comparing patients with mood disorders or anxiety and those without, there were no significant differences in PL DCDs, neither in other CI observed.

Among the autoantibodies tested, anti-phospholipid antibodies positivity was significantly associated with memory impairment (P<0.0005); moreover, the presence of other NP events was associated with executive dysfunctions (P<0.05). We didn’t find further association with mood and anxiety disorders, treatments (e.g. immunosuppressant, glucocorticoids dosage, antiplatelet and anticoagulant therapy), comorbidities (like dyslipidemia, hypertension, diabetes) demographical (such as age, education level), clinical and laboratory SLE features, including activity and damage indices (statistical analysis non reported due to space constraints). Table 3 summarizes the main clinical and demographic features of the cohort, divided in 2 groups, according the presence of PL dysfunction.

**Table 3:** Clinical and demographic features of SLE patients with and without PL dysfunction.

ACR criteria	PL dysfunction	Not PL dysfunction	P
	N/%	N/%	
Malar rash	6/33.3	15/68.2	NS
Discoid lupus	1/5.5	0	NS
Photosensitivity	7/38.8	10/45.4	NS
Mouth and nasal ulcers	16/88.8	13/59.1	NS
Arthritis	12/66.6	17/77.3	NS
Serositis	4/22.2	4/18.2	NS
Renal involvement	4/22.2	4/18.2	NS
Hematologic manifestations	7/38.8	13/59.1	NS
Neuropsychiatric involvement	2/11.1	3/13.6	NS
**Autoantibodies**			
Anti-dsDNA	12/66.6	17/77.3	NS
Anti-SSA	5/27.7	6/27.3	NS
Anti-SSB	1/5.5	3/13.6	NS
Anti-SM	2/11.1	4/18.2	NS
Anti-RNP	2/11.1	3/13.6	NS
Hypocomplementemia	9/50	10/45.4	NS
Anti-cardiolipin	5/27.7	8/36.4	NS
Anti B2GPI	4/22.2	7/31.8	NS
Lupus Anticoagulant	2/11.1	6/27.3	NS
**Therapy**			
Glucocorticoids	3/16.7	9/40.9	NS
Hydroxychloroquine	14/77	15/68.2	NS
Methotrexate	3/16.7	3/13.6	NS
Azathioprine	4/22.2	8/36.4	NS
Cyclosporine	2/11.1	3/13.6	NS
Mycophenolate mofetil	4/22.2	2/9	NS
Cyclophoshamide	1/5.5	0	NS
Belimumab	0	1/4.5	NS
Rituximab	1/5.5	1/4.5	NS
Antiplatelet therapy	5/27.7	7/31.8	NS
Anticoagulant treatment	1/5.5	1/4.5	NS

## Discussion

In the present study, for the first time we evaluated PL in a single center cohort of SLE patients, demonstrating a dysfunction in almost half of enrolled patients. Moreover, the impairment of this specific domain resulted significantly more frequent compared to the other domains assessed. In 1999 the ACR *ad hoc* committee defined cognitive dysfunctions as the impairment involving at least one of the following domains: simple or complex attention, memory (i.e. learning, recall), reasoning, problem solving, psychomotor dexterity, visuospatial abilities, language, executive functions (i.e. planning, organization, working memory, cognitive flexibility). The spectrum of cognitive dysfunctions can assume different severity (mild, moderate, severe) based on the number and intensity of involvement and of their impact on the ability to work or create social relations [1]. Moving on SLE patients, cognitive impairment could be observed regardless overt neuropsychiatric involvement, however a clear neurocognitive profile has not been defined yet. In a recently published literature review with meta-analysis, complex attention, delayed verbal memory, language and verbal reasoning were the most compromised domains in SLE patients [26]. Finally, the 10-years follow-up in a large SLE cohort demonstrated the improvement of CI in 50% of patients [27].

Of note, this systematic review highlighted a relatively limited assessment of language deficits in SLE and suggested the need of further investigations on this specific domain.

PL refers to social language skills used in daily interactions and requires integration of verbal message with reference framework. This specific impairment extensively assessed in brain traumatic injury, autistic spectrum disorders and right hemisphere damage, has been also reported in several adult diseases such as early Alzheimer’s, Parkinson’s disease and even multiple sclerosis [28,29,30]. Diagnosis of CI in SLE is generally delayed because of the lack of proper screening and diagnostic tools [31]. Moreover, ACR recommended as gold standard tool a time-consuming battery, limiting the applicability of its administration in clinical practice [32]. Unfortunately, this tool is not able to assess PL and an impairment in non-literal language could be difficult to identify without proper test. Neuro-psychological batteries have been developed to assess PL. In particular, in English speaking countries the Right Hemisphere Communication Battery [33] and Clinical Management of Right Hemisphere Dysfunction [34] has been the most frequently cognitive tests used so far. Similarly, BLED battery has been developed by Italian institute Santa Lucia: it is specifically focused on diagnosis and clinical management of PL dysfunction [25].

In our cohort, a deficit of PL was observed in almost half of patients; interestingly, this dysfunction was significantly more prevalent than other frequently involved domains, such as memory, executive and visuospatial functions. The lack of association with other disease-related factors could suggest a specific pathogenic mechanism, not identified so far and the need to assess PL regardless other cognitive domains impairment, including executive functions. Nowadays there is no evidence that this peculiar dysfunction could be autoantibody mediated, even more we didn’t find any statistically significant association with ENA, anti-dsDNA and antiphospholipid antibodies.

In addition, when considering the global cognitive functions, a mild impairment was found in 25% of SLE patients, and moderate in 7.5%, according with low age and high education level. Despite the high prevalence of mood disorders, the analysis for confounders ruled out any effect on cognitive dysfunctions assessed in our cohort. Moreover, we confirmed the association between SLE-related neuropsychiatric involvement and aPL positivity, specifically regarding to executive functions and memory domains.

At the best of our knowledge this is the first study investigating PL impairment in SLE patients and in other systemic autoimmune diseases.

In conclusion, the results of the present study demonstrate that PL dysfunction is not only prevalent but can be also severe in SLE patients, suggesting the possibility of an evaluation of this specific domain.

## Limits of the study

Our cohort showed a low disease activity and damage scores and only 30% of patients enrolled were on glucocorticoids treatment. Even if we could assume that treatment didn’t influenced CI in our patients, we would highlight that neurocognitive impairment is generally associated to disease activity [35]. Moreover we would point out that the absence of a disease control group could be a limit due the difficulty to confirm that PL dysfunction is specifically provoked by SLE. Finally we did not perform any imaging evaluation in this study. We believe that PL assessment should be performed in other SLE cohort, especially those different for language and culture, in order to confirm our results.

## Supporting information

S1 File The datasets used and/or analyzed during the current study are available as supplementary file. (XLSX)
